# Biochemical and genetic analysis of the role of the viral polymerase in enterovirus recombination

**DOI:** 10.1093/nar/gkw567

**Published:** 2016-06-17

**Authors:** Andrew Woodman, Jamie J. Arnold, Craig E. Cameron, David J. Evans

**Affiliations:** 1 Biomedical Sciences Research Complex, North Haugh, University of St. Andrews, St. Andrews KY16 9ST, UK; 2 Dept. of Biochemistry & Molecular Biology, 201 Althouse Lab, University Park, PA 16802, USA

## Abstract

Genetic recombination in single-strand, positive-sense RNA viruses is a poorly understand mechanism responsible for generating extensive genetic change and novel phenotypes. By moving a critical *cis*-acting replication element (CRE) from the polyprotein coding region to the 3′ non-coding region we have further developed a cell-based assay (the 3′CRE-REP assay) to yield recombinants throughout the non-structural coding region of poliovirus from dually transfected cells. We have additionally developed a defined biochemical assay in which the only protein present is the poliovirus RNA dependent RNA polymerase (RdRp), which recapitulates the strand transfer events of the recombination process. We have used both assays to investigate the role of the polymerase fidelity and nucleotide turnover rates in recombination. Our results, of both poliovirus intertypic and intratypic recombination in the CRE-REP assay and using a range of polymerase variants in the biochemical assay, demonstrate that RdRp fidelity is a fundamental determinant of recombination frequency. High fidelity polymerases exhibit reduced recombination and low fidelity polymerases exhibit increased recombination in both assays. These studies provide the basis for the analysis of poliovirus recombination throughout the non-structural region of the virus genome and provide a defined biochemical assay to further dissect this important evolutionary process.

## INTRODUCTION

Recombination in single-stranded, positive-sense RNA viruses is a relatively poorly understood driver of extensive genetic change. This group of viruses, typified by poliovirus, exist as viral quasispecies as a consequence of misincorporations by their error-prone RNA dependent RNA polymerases (RdRp) during genome replication coupled with strand transfer events that create hybrids (recombinants) between two viruses replicating in the same cell. As well as being a driver of genetic variation, recombination may have evolved to ‘rescue’ genomes from deleterious mutations that accumulate during error-prone replication (1) though this may only be important for small population sizes such as might occur during inter-host transmission (2).

Poliovirus, the prototype picornavirus and aetiological agent of paralytic poliomyelitis, provides a tractable experimental system to study recombination. The 7.5 kb genome encodes a single polyprotein, flanked by non-coding regions (NCR), that is co- and post-translationally processed to generate the structural proteins (the P1 proteins; VP4, VP2, VP3 and VP1) which assemble to form the icosahedral capsid and the non-structural proteins (the P2 and P3 proteins; 2A^pro^, 2B, 2C, 3A, 3B^VPg^, 3C^pro^ and 3D^pol^) that subvert the cellular environment and replicate the virus genome. The genome can essentially be considered modular, consisting of structural (P1) and replication (P2 and P3) components, together with the flanking translation and replication determinants occupying the 5′ and 3′ NCRs (3,4). This modularity is emphasized in *in vitro* studies in which viable recombinants have been engineered or selected (5,6). More compellingly, studies of paralysis induced by vaccine derived recombinant polioviruses (VDRP)—in which the live attenuated Sabin vaccine strain has recombined with a co-circulating species C enterovirus—have demonstrated the importance of recombination *in vivo* (7). In related enteroviruses, recombinant forms—defined by serotype according to their capsid proteins—have been shown to emerge, prevail and then disappear in temporal epidemiological surveys of globally-distributed serotypes (8–10). In these examples the recombinants are pathogenic (generally associated with poliomyelitis, a range of acute flaccid paralyses or viral encephalitis) and demonstrate maintenance and transmission of the capsid in the population by a range of non-structural ‘modules’ from genetically-related but divergent viruses. An improved mechanistic understanding of recombination is required, both to comprehend the process as a driver of evolutionary change and to develop strategies to prevent or mitigate the consequences of recombination, for example by the design of non-recombinogenic live-attenuated vaccines.

We have recently described an *in vitro* assay (designated the CRE-REP assay) that enabled the identification of early recombination products (11). Briefly, the assay involves two poliovirus genomes each containing a different deleterious (and non-reverting) modification that prevents the production of viable progeny. One genome, a sub-genomic replicon (12), was replication competent but did not encode capsid proteins. The other contained a well-understood mutation (designated SL3) in a critical *cis*-acting replication element (CRE), a defined stemloop structure essential for positive-strand replication (13,14). Co-transfection of murine cells—permissive for infection, but not-susceptible due to the absence of the poliovirus receptor —resulted in the recovery of viable progeny following a recombination strand transfer event between the non-structural protein (P2 and P3) modules of the sub-genomic replicon (the polymerase donor) and the structural (P1) module of the CRE-defective SL3 genome. By using genomes of different poliovirus serotypes with divergent sequences it was possible to unambiguously identify the recombination junction. To our surprise, the majority of initial viable recombinants were ‘imprecise’ and contained an in-frame partial genome duplication. Subsequent serial passage resulted in the incremental loss of the genome duplication, leaving a range of ‘precise’ junctions with no additional sequences (11). These observations strongly suggest that recombination may be biphasic, involving the formation of an initial imprecise product that, through a process we termed resolution, yields the genome-length recombinants that are typically isolated *in vivo*. Further studies demonstrated that modifying the polymerase fidelity significantly influenced the yield of recombinants, an observation supported by a recent study in alphaviruses (15). This indicated both a fundamental role for the polymerase in the process of recombination and that the process was replicative and therefore different from previously described non-replicative recombination (16,17).

Although the CRE-REP assay enables the role of the polymerase in recombination to be analysed it does not demonstrate that the polymerase is sufficient for strand exchange. Furthermore, although modification of the polymerase fidelity influences recombination yield in the CRE-REP assay, this does not exclude the possibility that this is an indirect consequence of the polymerase interacting with cellular components of the replication machinery. Recombination occurs within membrane-bound replication complexes (RCs), as shown by elegant imaging studies and the demonstration that nocodazole—a drug that prevents RC coalescence—inhibits the process (11,18). Since the components of the RC are not fully understood we reasoned that a defined *in vitro* recombination assay would enable the role of viral, and possibly cellular, components to be more readily delineated. Using such an assay we demonstrate here that the viral polymerase alone is sufficient for the strand transfer reaction. In addition, by analysis of variants of the polymerase with well-characterised changes to the enzymes’ fidelity or nucleotide turnover, we demonstrate both that this biochemically defined *in vitro* assay recapitulates aspects of the CRE-REP assay and that the fidelity of the viral polymerase is a key determinant of the recombination process. In support of this conclusion we demonstrate that the majority of *in vitro*-generated transfer products contain additional nucleotides or mutations at or near the recombination junction. By modifying components of the *in vitro* assay we show that the strand transfer event is sequence-dependent. Finally, we modified the CRE-REP assay and demonstrate that recombinant yield is influenced by the distance between the deleterious mutations in the parental genomes and can partially compensate for polymerases with reduced nucleotide turnover. These studies will enable the analysis of recombination events throughout the region encoding the non-structural proteins of poliovirus, and—by extrapolation—to related enteroviruses and other positive sense RNA viruses. This study provides the basis for the detailed kinetic and mechanistic analysis of the initial strand-transfer and subsequent resolution events critical for the formation of both viable and competitively fit recombinant viruses.

## MATERIALS AND METHODS

### Viruses and cell culture

Adherent monolayers of HeLa and L929 fibroblasts were grown in Dulbecco's Modified Eagle Medium (DMEM) or Glasgow Minimum Essential Medium (GMEM supplemented with G418 antibiotic). Media was supplemented with 100 U/ml penicillin, 100 μg/ml streptomycin, 2 mM l-glutamine and 10% heat inactivated (HI)-FBS. All cells were passaged in the presence of trypsin–EDTA. Where stated, guanidine hydrochloride (Sigma) was added to growth media at 4 mM. Poliovirus type 1 (Mahoney) and type 3 (Leon) were recovered following transfection of RNA generated *in vitro* (see below) from full-length cDNA. Virus was quantified by plaque assay or TCID_50_ as appropriate and as described previously (19). Virus growth analysis was determined by synchronous infection of HeLa cells at a multiplicity of infection (moi) of 10 pfu/cell, washing with PBS to remove unadsorbed virus and incubation in fresh media at 37°C in an atmosphere containing 5% CO_2_. Supernatant virus was quantified at various time points post infection by plaque assay. Virus competition assays were conducted by co-infection (at the specified ratios) of HeLa cells with a final moi of 10 pfu/cell. When serially passaging virus, harvested supernatant was diluted 1:4 with fresh media.

### Plasmids, *in vitro* transcription, cell transfection and recombinant virus characterisation

pRLucWT and pT7Rep3-L are, respectively, cDNAs in pBR-derived plasmids encoding poliovirus type 1 Mahoney and type 3 Leon sub-genomic replicons with a luciferase reporter gene inserted in-frame in place of the P1 capsid coding region (13,20). pRLucWT_G64S_ contains a Gly to Ser substitution at residue 64 of the viral polymerase. Derivatives of these replicons bearing substitution of lysine 359 with arginine (pRLucWT_K359R_ and pT7Rep3-L_K359R_) were constructed using standard molecular protocols and verified by sequencing. pT7/SL3 has been described previously (13) and consists of a full-length poliovirus type 3 (Leon) cDNA bearing 8 synonymous substitutions in the *cis*-acting replication element (CRE) in the 2C-coding region. pT7/SL3_K359R_ was constructed by exchange of a relevant restriction fragment from pT7Rep3-L_K359R_ into pT7/FLC, a plasmid carrying a full length cDNA for poliovirus type 3 Leon. A derivative of pRLucWT deleted for the CRE in the native location (pRLucWTΔCRE) was generated by site directed mutagenesis introducing the same 8 substitutions defined in the SL3 mutant of poliovirus previously described (13). A synthetic CRE was introduced into the region encoding the 3′ NCR of pRLucWTΔCRE by first introducing a *Bss* HII site immediately following the site of polyprotein termination and then adding complementary oligonucleotides for the Synth2 version of the CRE (21) so creating pRLucWTΔCRE_3′CRE. Similar methods were used to introduce a 3′CRE into a full length poliovirus type 3 genome to create pFLCΔCRE_3′CRE.

Plasmids encoding type 1 and type 3 poliovirus genomes (full length or sub-genomic) were linearized with *Sal* I and *Apa* I respectively, transcribed *in vitro* using T7 RNA Polymerase (Fermentas), treated with 2 U DNAse Turbo (Ambion) to remove residual DNA template and the RNA transcripts purified using RNeasy Mini Kit (Qiagen) before spectrophotometric quantification. Unless otherwise specified 1μg of RNA was transfected into near-confluent T25 flasks using Lipofectamine 2000 (Invitrogen).

Recovered recombinant viruses were isolated by limit dilution and sequenced following RT-PCR amplification across the recombination junction as described previously (11). Viral RNA was extracted from clarified culture supernatant using a Qiagen RNAeasy Mini kit, reverse transcribed using Superscript II reverse transcriptase (Invitrogen) and an oligo-dT primer at 46°C for 50 min with the reaction terminated by incubation for 15 min at 70°C. PCR amplification of recombination junctions used template cDNA and appropriate oligonucleotides as listed in Supplementary Table T1 with KOD XL DNA polymerase (Novagen) used according to the manufacturer's protocol. Growth competition studies between parental type 3 poliovirus and FLCΔCRE_3′CRE were conducted by coinfection at a final moi of 10pfu/cell using 1:1, 10:1 and 1:10 ratios of input, with recovered virus being RT-PCR amplified across the region encoding the 2C-CRE followed by *Swa* I digestion of the PCR product and agarose gel electrophoresis.

### Luciferase assays

Supernatant was removed from transfected cell monolayers, cells were briefly washed with PBS and lysed using 200 μl 1× Glo Lysis Buffer (Promega^®^) per well in a 12-well plate. The oxidation reaction was catalysed by the addition of 50 μl cell lysate to 50 μl room temperature *Bright-Glo*TM Luciferase Assay System (Promega^®^) substrate and shielded from the light for 5 mins. Luciferase activity was measured using a luminometer with values normalised to mock transfection controls.

### *In vitro* sym/sub-based template switching assay

The sym/sub assay has been described previously (22). Elongation complexes were assembled by incubating 5 μM WT or mutant poliovirus polymerase with 1 μM sym/sub RNA primer-template and 500 μM ATP for 5 min (Mix 1). Unless otherwise specified, template-switching reactions were initiated by addition of 60 μM RNA acceptor template and 500 μM CTP, GTP and UTP (Mix 2) and then quenched at various times by addition of 50 mM EDTA. All reactions were performed at 30°C in 50 mM HEPES, pH 7.5, 10 mM 2-mercaptoethanol, 60 μM ZnCl_2_, and 5 mM MgCl_2_. Products were analyzed by denaturing polyacrylamide gel electrophoresis, visualized using a PhosphorImager and the transfer products quantified using ImageQuant software (GE Healthcare). The strand transfer product was excised from the gel and sequenced following the miRCat-33™ protocol (Integrated DNA Technologies).

### CRE-REP recombination assay

The CRE-REP assay for replicative recombination was conducted as described previously (11) and similar conditions were used for the 3′CRE-REP assay developed during this study. Where specified, ribavirin was added to transfected L929 monolayers at 600 μM. Recombinant viruses were quantified by plaque assay.

## RESULTS

### Polymerase fidelity and nucleotide turnover influences viral recombination

Mutations to the viral polymerase can influence the fidelity and nucleotide turnover rate of the enzyme. Previous studies have demonstrated that substitution of a glycine to serine at position 64 in the poliovirus polymerase (G64S) confers resistance to ribavirin, a guanine-analog (23,24). We have demonstrated that this mutation decreased recombinant yield in an intertypic (poliovirus type 1 versus poliovirus type 3) CRE-REP assay by ∼20-fold whereas the addition of ribavirin, which enhances the polymerase error rate, increased recombinant yield in the same assay (11). To determine whether independently identified polymerase fidelity variants similarly influenced recombination we investigated the consequences of a conserved lysine to arginine substitution at residue 359 (K359R). This residue, located within motif D of the polymerase adjacent to the active site, is proposed to act as the general acid required for the second protonation event during polymerase-catalyzed nucleotidyl transfer (25). Polymerases bearing a K359R substitution retain activity but turn over nucleotides at 5–10% the rate of the unmodified protein and are both genetically-stable and attenuating (26). We additionally studied the influence of a polymerase with an extensively characterized (27) mutator phenotype, bearing a histidine to arginine substitution at residue 273 (H273R). This polymerase substitution has no impact on virus replication (27) but increases the mutation rate 3-fold and markedly attenuates the virus *in vivo*. Similar low fidelity polymerases have recently been shown to positively influence the yield of recombinant and defective interfering (DI) virus in an alphavirus model (15).

**Figure 1.**
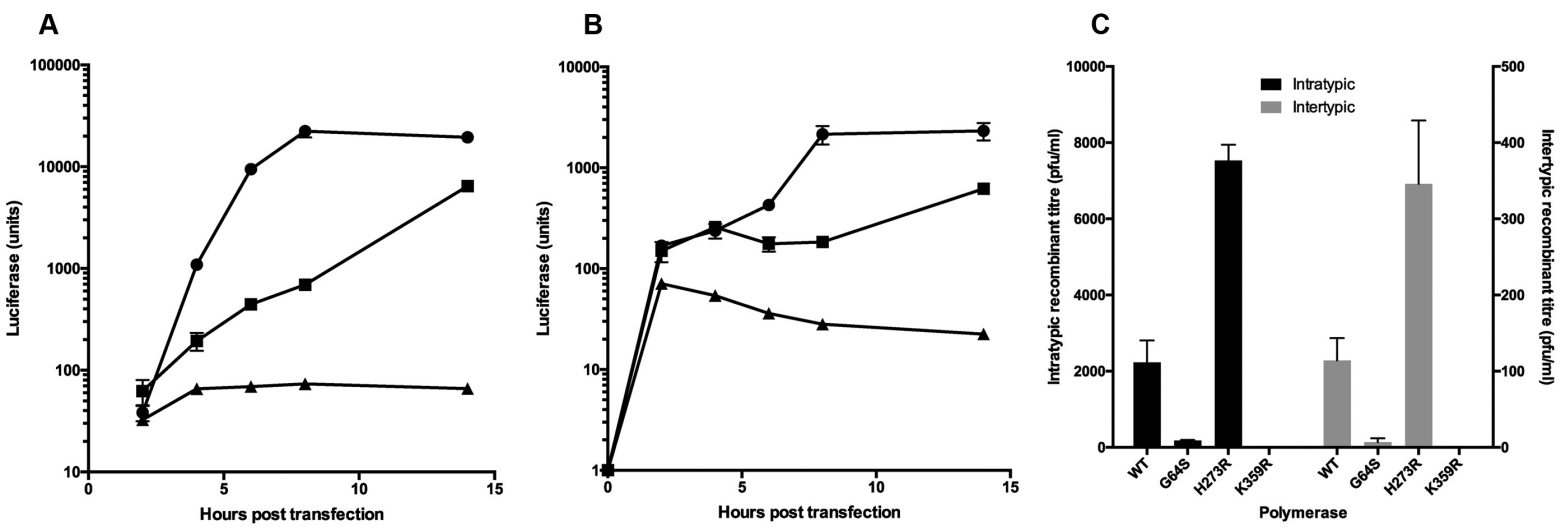
Replication and recombination analysis of poliovirus bearing a K359R mutation in the viral polymerase. (**A** and **B**) Replication kinetics of sub-genomic replicons of poliovirus type 1 (RLucWT – A) or poliovirus type 3 (Rep3-L – B). Unmodified (wild-type) replicons indicated with filled circles, replicons bearing a K359R polymerase mutation with filled squares and wild-type replicons in the presence of 4mM guanidine hydrochloride, an inhibitor of poliovirus RNA replication, with filled triangles. 250ng of RNA generated *in vitro* was transfected into HeLa cells. Samples were taken at the times indicated and luciferase activity was measured and normalised using a mock transfected control. Error bars indicate standard deviation of two independent samples. (C) Influence of G64S, H273R and K359R polymerase mutants on the yield of recombinants in an intertypic (grey bars) and intratypic (black bars) CRE-REP recombination assay. Yield of recombinants is expressed as pfu/ml with error bars showing standard deviation from at least three independent assays. Note that the intratypic and intertypic assays use different scale Y axes. The X axis indicates the identity of the viral polymerase in both donor (sub-genomic replicon) and acceptor (SL3-derived genome) templates. Plaque assay quantities were independently confirmed by TCID_50_ (data not shown).

We engineered the K359R or H273R substitutions into poliovirus type 1 and type 3 sub-genomic replicons and quantified luciferase production after transfection of HeLa cells with RNA synthesised *in vitro*. Both K359R-containing replicons exhibited a marked delay in maximal luciferase synthesis and a lower overall yield, reaching ∼30% of the luciferase level generated by the unmodified parental replicons (Figure 1A and B). We additionally engineered the K359R or H273R substitutions into the poliovirus type 3-derived SL3 genome, generating SL3^K359R^ and SL3^H273R^ respectively. We then conducted independent CRE-REP recombination assays using the SL3^K359R^ and SL3^H273R^ acceptor genomes with type 1 or type 3 sub-genomic replicons bearing the matching mutation, monitoring the yield of inter- and intratypic recombinants produced in 48 hours following transfection of murine L929 cells.

Using unmodified (WT) parental SL3 and sub-genomic replicons yielded ∼2200 pfu/ml (±571; all titres determined in three or more independent assays and expressed ± SD) and 114 pfu/ml (±29) in intratypic and intertypic CRE-REP assays respectively, in broad agreement with previous results (11). In parallel assays the previously characterized G64S high fidelity polymerase variant produced 180 pfu/ml (±14) and 7 pfu/ml (±5) in intra- and intertypic recombinants respectively, the latter in agreement with earlier results (11). In the intertypic CRE-REP assay, genomes bearing the H273R substitution yielded 346 pfu/ml (±83; Figure 1C), an increase of ∼3-fold over the unmodified templates. A comparable (∼3.3-fold) increase in recombinant yield was also observed in the intratypic CRE-REP assay with templates bearing the same H273R variant polymerase. In contrast, the presence of the K359R mutation in both donor and recipient genomes prevented the recovery of any viable recombinants in either inter- or intratypic CRE-REP assays (Figure 1C). The results obtained with the low fidelity H273R polymerase, together with the recombination phenotype of the high fidelity K359R and G64S (11) enzymes, strongly support a role for polymerase fidelity as a key determinant of recombination frequency.

### Engineering the CRE into the 3′ non-coding region of the poliovirus genome

The frequency of recombination is presumably related to the opportunity the polymerase has to switch templates. For example, in previous studies we demonstrated that preventing replication complex coalescence (via nocodazole inhibition of microtubule polymerisation) reduced the yield of recombinants. Since the generation of viable recombinant progeny in the CRE-REP assay is dependent upon a strand transfer event occurring within the ∼1kb separating the P1-coding region of the sub-genomic replicon and the defective CRE in the SL3 acceptor template (11) we reasoned that increasing the separation of these selection markers should also increase recombinant yield which would confer advantages in analysis of polymerases with reduced nucleotide turnover, such as K359R.

**Figure 2.**
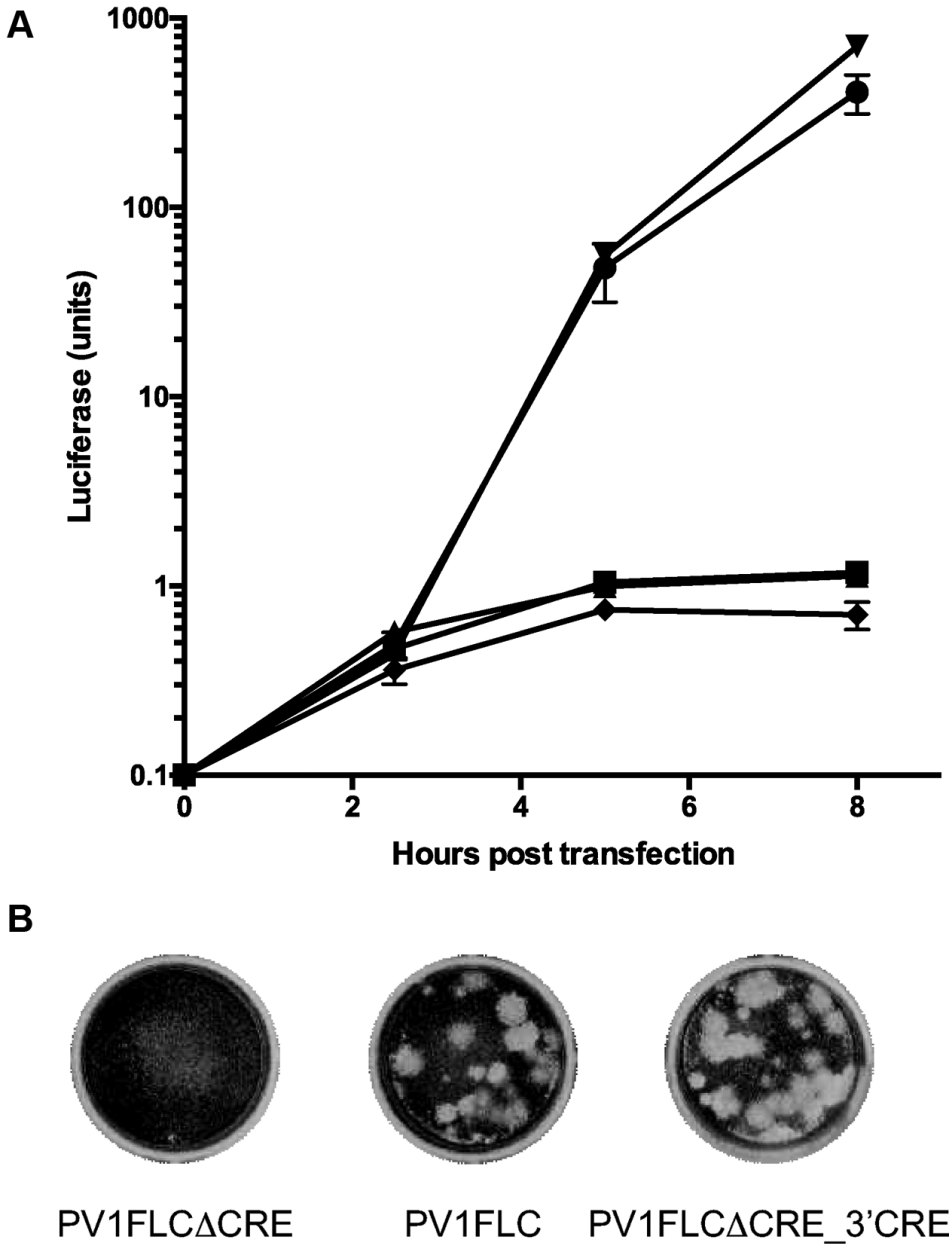
Replication and recombination of poliovirus with the CRE located in the 3′ non-coding region. (**A**) Replication of sub-genomic poliovirus type 1 replicons expressing a luciferase reporter gene. Wild-type (RLucWT) replicon in the absence (•) and presence (▴) of 4mM guanidine hydrochloride, together with the replicon with the native CRE inactivated (RLucWTΔCRE; ♦). The luciferase activity expressed by a replicon with a synthetic CRE inserted into the 3′ NCR (RLucWTΔCRE_3′CRE) in the absence (▾) and presence of 4mM guanidine hydrochloride (▪). In each case, 250ng of relevant RNA transcribed *in vitro* was transfected into L929 cells in duplicate, samples were harvested at the time points indicated and luciferase activity was measured and normalized using a mock transfected control. (**B**) Representative crystal violet stained Hela cell monolayers inoculated with supernatant obtained following transfection of RNA transcribed *in vitro* from the relevant full-length cDNAs indicated. Infected HeLa cells were covered with plaque assay overlay medium and stained 3 days post infection.

Since the majority of characterised naturally-occurring recombinant enteroviruses exhibit recombination junctions within the P2- or P3-coding regions of the genome we wanted to modify the CRE-REP system to allow analysis of recombinants generated *in vitro* within a similar region. We therefore altered the original poliovirus type 1 sub-genomic replicon (pRLucWT) to inactivate the native CRE in the 2C coding region by the introduction of 8 synonymous substitutions (identical to those used in the construction of the SL3 variants used here and previously; (13)) to generate pRLucWTΔCRE. We confirmed that, as expected, RNA synthesized *in vitro* from pRLucWTΔCRE and transfected into murine L929 cells could not replicate (Figure 2A). The luciferase signal generated was similar in the presence or absence of 4 mM guanidine hydrochloride, a potent inhibitor of poliovirus replication (28), indicating it was solely from translation of the transfected RNA. We subsequently engineered a synthetic variant of the CRE sequence into the 3′ NCR of a pRLucWTΔCRE to generate pRLucWTΔCRE_3′CRE. The synthetic CRE sequence used had previously been shown to function when inserted into the 5′ NCR and consisted of the terminal 18 nt. of the wild-type CRE sequence engineered to form the terminal loop on a stem with similar structural stability to the native element (Supplementary Figure S1). This sequence was known to be stable in genomes bearing dual CRE sequences (21). RNA generated *in vitro* from pRLucWTΔCRE_3′CRE was transfected into murine L929 cells and luciferase synthesis monitored for 8 hours post-transfection. In parallel assays the luciferase signal from pRLucWTΔCRE_3′CRE-derived RNA was marginally greater than that from RNA generated from the parental sub-genomic replicon, pRLucWT, confirming that insertion of the CRE within the 3′ NCR was not incompatible with genome replication (Figure 2A).

To ensure that the resulting modifications had not introduced unexpected functional defects (e.g. disruption of encapsidation, a process critical for the identification of viable recombinants in the CRE-REP assay) into the virus genome we engineered the same mutations to the native CRE (creating pPV1FLCΔCRE) and 3′ NCR (creating pPV1FLCΔCRE_3′CRE) in a full length infectious poliovirus type 1 cDNA (pPV1FLC). RNA generated *in vitro* was independently transfected into murine L929 cell monolayers and *de novo* generated supernatant virus was quantified by subsequent plaque assay on HeLa cells. pPV1FLCΔCRE yielded no measurable virus and subsequent blind serial passage did not lead to the emergence of variants capable of replicating, a result in agreement with comparable studies on poliovirus type 3 (13). In contrast, RNA from pPV1FLCΔCRE_3′CRE generated viral plaques indistinguishable in appearance from the parental unmodified cDNA (Figure 2B) with a yield of 6 × 10^8^ pfu/ml, similar to the 4 × 10^8^ pfu/ml yield from PV1FLC under similar assay conditions. To further confirm that a virus bearing the CRE in the 3′ NCR replicated with similar kinetics to the unmodified parental virus we conducted competition experiments between the two viruses, broadly recapitulating the conditions that would prevail in a mixed infection recombination assay (Supplementary Figure S2). The results demonstrated that the poliovirus CRE can be relocated to the 3′ NCR without apparently compromising virus replication, thereby making it a suitable template for analysis of recombination throughout the region encoding the virus non-structural proteins.

### Extending the CRE-REP assay

**Figure 3.**
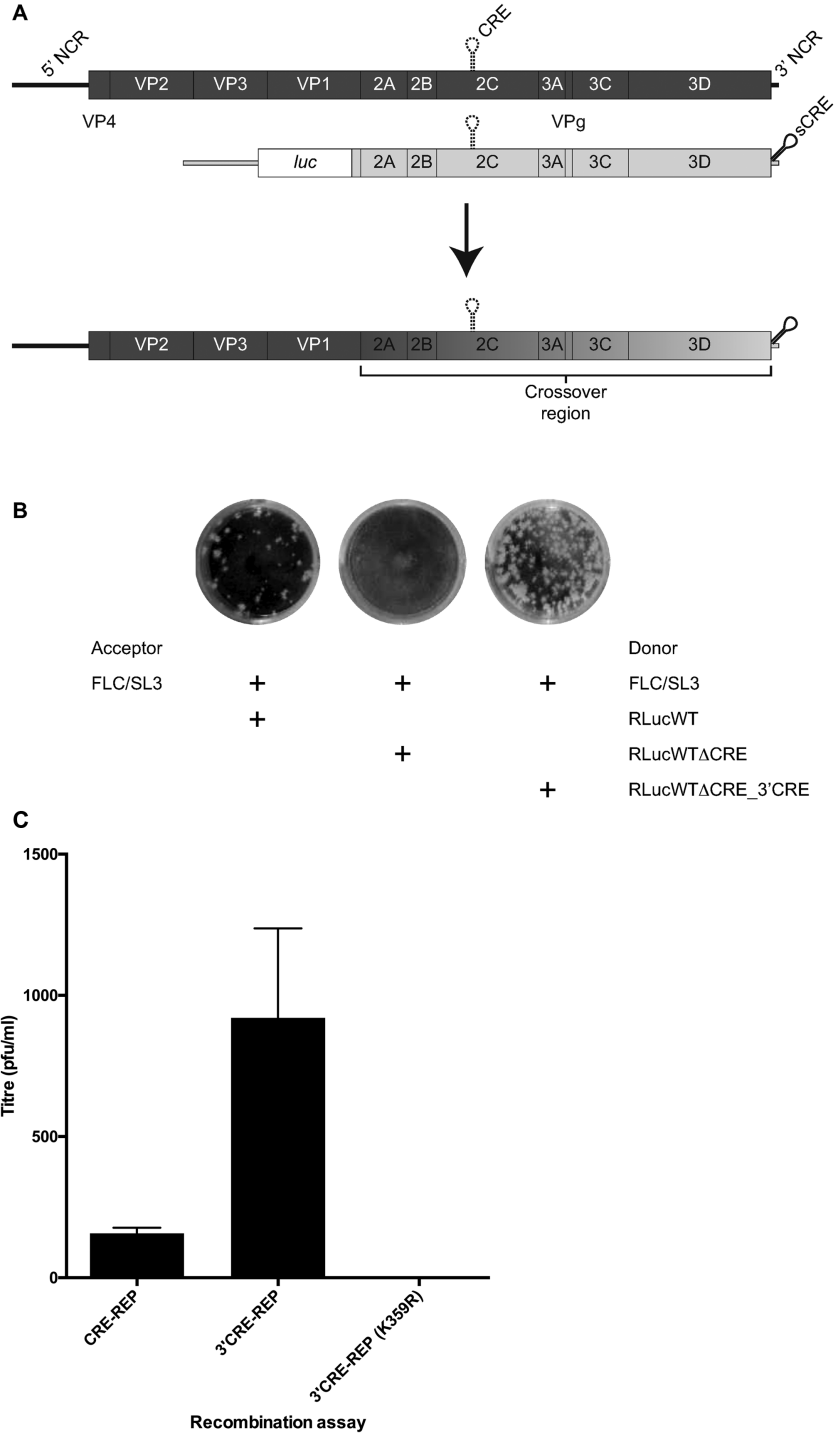
An extended 3′CRE-REP recombination assay. (**A**) Schematic depiction of the 3′CRE-REP recombination assay. The acceptor genome (dark shading) bearing a defective CRE indicated as a broken line in the 2C-coding region is shown above a representation of the donor genome, a luciferase-encoding sub-genomic replicon (light shading) in which the native CRE has been inactivated and a synthetic CRE (indicated sCRE) inserted into the 3′ NCR. Following co-transfection of permissive cells (indicated by an arrow), a replication competent recombinant genome may be recovered of the generic structure shown, consisting of the 5′ part of the genome derived from full-length, capsid-encoding, acceptor genome and the 3′ part from the luciferase-encoding donor replicon. The crossover may occur within the region indicated. (**B**) Increased recombinant yields from the 3′CRE-REP assay. 250 ng of the acceptor or each of the indicated donor RNAs were co-transfected into L929 murine cells and supernatant harvested at 48 h post-transfection. Recombinant virus present in similar dilutions of supernatant was compared by plaque assay in HeLa cells and stained 72 h post infection. (**C**) Comparison of intratypic recombination yields from CRE-REP and 3′CRE-REP assays primed with RLucWT and FLC/SL3 donor and acceptor genomes. The presence of K359R polymerase mutation in both donor and acceptor genomes prevented the generation of recombinants in the 3′CRE-REP assay. The data represents the mean from three independent samples (± standard deviation). All plaque assay results were confirmed by TCID_50_ (data not shown).

The relocated CRE in the 3′ NCR is situated ∼2.9 kb from it's native position, extending the potential region for recombination—the distance separating the VP1 capsid-coding region (absent from the sub-genomic replicon) and a functional CRE (absent from the SL3 parental genome)—from ∼1 kb to almost 4 kb (Figure 3A). We investigated intertypic recombination following transfection of murine L929 cells with RNA generated *in vitro* from pRLucWTΔCRE_3′CRE and pT7FLC/SL3. In parallel, we conducted a control CRE-REP recombination assay with RNA derived from parental pRLucWT and pT7FLC/SL3 (Figure 3B). The latter generated 157 pfu/ml (±20) whereas the assay with the extended recombination region yielded an increase of ∼5.8-fold to an average of 920 pfu/ml (±317; Figure 3C). Preliminary analysis of recombination junctions in viruses recovered in the extended CRE-REP assay (henceforth designated 3′CRE-REP) indicated they were located throughout the region encoding the non-structural proteins, with both imprecise and precise junctions identified (Supplementary Figure S3).

We then used the 3′CRE-REP assay to investigate whether the increased yield of recombinants was sufficient to detect recombination in genomes bearing the K359R mutation. Following transfection of RNA derived from pRLucWTΔCRE_3′CRE and pT7FLC/SL3, with both cDNAs modified to include the K359R mutation in the 3D^pol^ coding region, no recombinants were recovered (Figure 3C). Therefore, although increasing the size of the region in which recombination could occur enhanced the yield of recombinants, it was not sufficient to compensate for the non-recombinogenic K359R mutation in the viral polymerase.

### A biochemically-defined recombination assay

**Figure 4.**
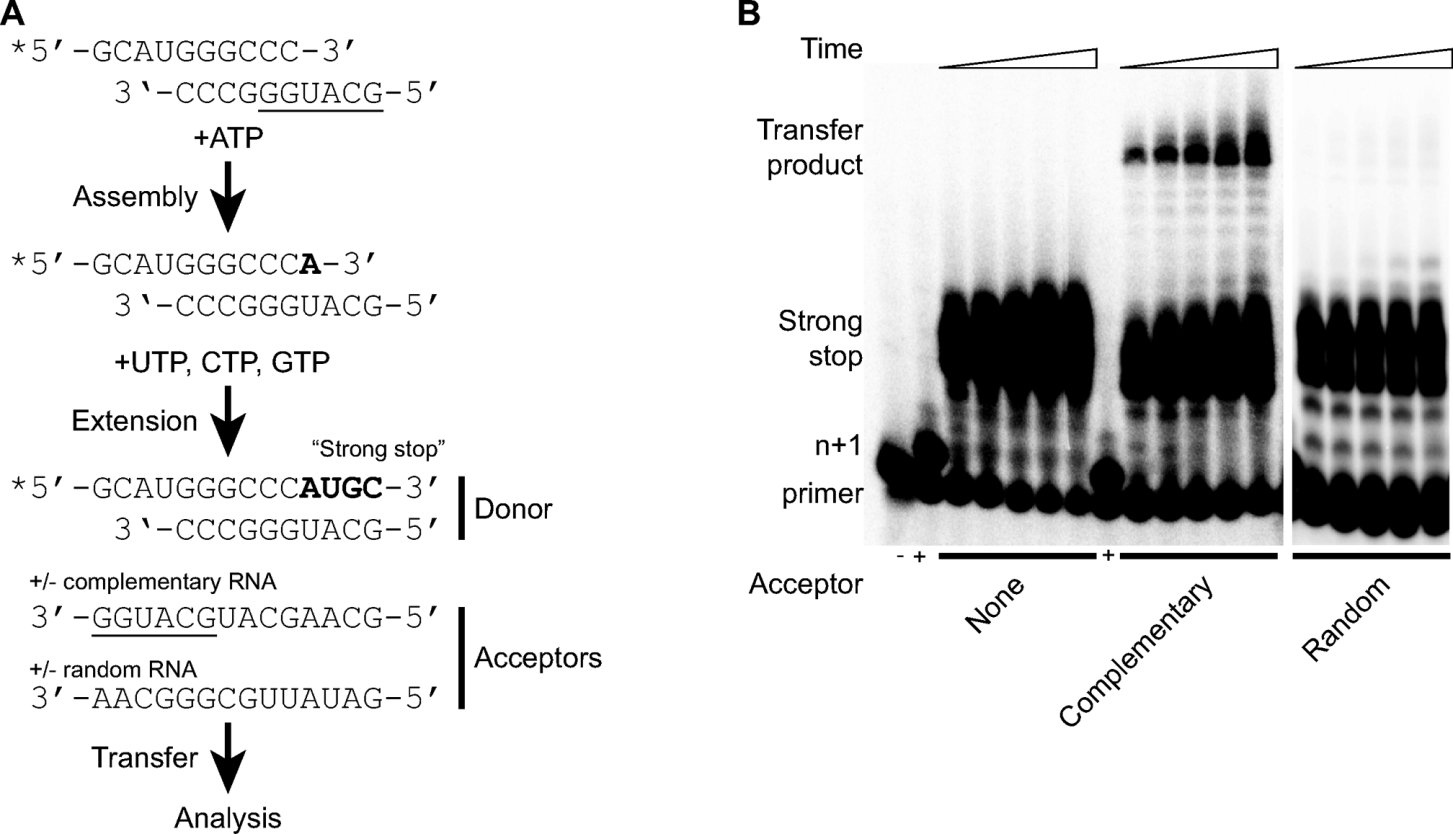
A biochemically-defined *in vitro* recombination assay. (**A**) Scheme for *in vitro* template-switching assay. A radiolabelled (end labelled, indicated *) primer substrate pair known as sym/sub is the basis for the assembly of an elongation complex by the addition of ATP and purified polymerase protein. Addition of remaining nucleotides allows complete extension of the sym/sub template to form a strong stop product. The latter is considered the donor template to which acceptor template ribo-oligonucleotides are added. The complementary acceptor has six nucleotides (underlined) that have the potential to pair with the 3′ end of the extended sym/sub donor product. An alternative acceptor template with no complementarity was also tested. The products of the assay are analysed by polyacrylamide gel electrophoresis (PAGE). (**B**) Denaturing PAGE of *in vitro* reactions quenched at 5, 10, 20, 40 and 60 min after addition of the UTP/CTP/GTP and acceptor template (indicated below) mix. Reactions conducted in the absence (-) or presence (+) of ATP only indicated. The *n* + 1 product is the assembled elongation complex. The strong stop product is the fully extended sym/sub template. The high molecular weight products result from a template switch to the indicated RNA acceptor.

To further investigate the strand-transfer reaction that must occur during recombination we exploited a biochemically-defined assay based upon the sym/sub system (22), a 10 nt. heteropolymeric RNA primer-template symmetrical self-complementary substrate (sym/sub) forming a 6 nt duplex flanked with 4 nt. 5′ unpaired regions (Figure 4A). Previous studies using homopolymeric templates and primers has suggested that purified poliovirus polymerase was sufficient for the strand-transfer event (29). We therefore investigated modification of this well-characterised sym/sub assay by inclusion of an acceptor template containing two potential regions of limited complementarity with the primary extension product. Briefly, the two-stage reaction involves assembling the radiolabeled sym/sub template with purified polymerase and ATP (Mix 1) in a suitable buffer to allow the formation of the elongation complex. Addition of the remaining nucleotides and an acceptor template (Mix 2) enables extension and—potentially—strand transfer, with the reaction products over a time course analysed by polyacrylamide gel electrophoresis. The formation of an *n* + 1 product indicates functional assembly of the elongation complex (Figure 4A and B) prior to the addition of Mix 2. This results in the rapid formation of a number of >*n* + 1 products including the fully extended sym/sub template represented by the *n* + 4 product (‘Strong stop’, Figure 4A and B). The generation of an *n* + 5 product is due to the non-templated addition to the end of the primer template, as previously described (29). The strand transfer product is represented by a discrete band appearing after the addition of Mix 2, the level of which increased as the reaction was incubated for up to 60 min (Figure 4B). Quantification of the strand transfer product at 30 min allowed the optimization of experimental conditions, including polymerase and acceptor template concentrations (Arnold *et al*., manuscript in preparation).

**Figure 5.**
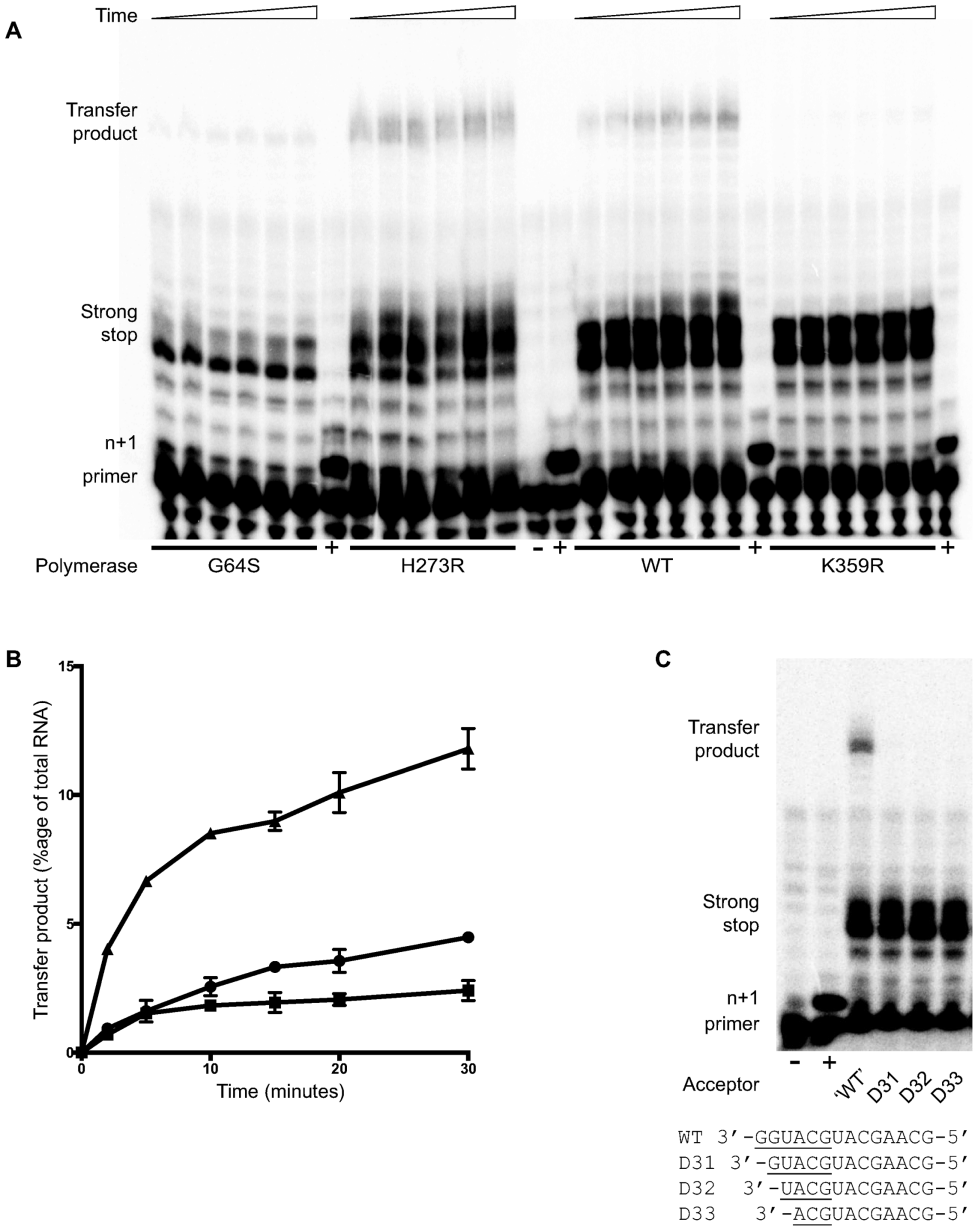
The influence of polymerase fidelity and acceptor template sequence. (**A**) Representative sym/sub template switching assays using the poliovirus polymerase variants indicated. Denaturing PAGE was used to separate reaction products at 2, 4, 8, 10, 20 and 30 min after addition of the extension mix. (**B**) Quantification of the transfer product generated by WT (•), H273R (▴) and G64S (▪) polymerases as a percentage of total RNA products per sample over time. Results are the average from three independent experiments and error bars indicate standard deviation. (**C**) Denaturing PAGE gel of *in vitro* sym/sub reactions quenched at 30 minutes after the addition of the extension mix containing complementary (WT) or acceptor RNAs truncated at the 3′ end by one, two, or three nucleotides (D31, D32 and D33 respectively) as shown.

Under the optimized conditions a maximum yield of ∼5% of the strand transfer product was achieved after 30 min when catalyzed by the native virus RdRp at 5 μM (Figures 4B and 5B). We reasoned that the acceptor template needed to be present in molar excess over the sym/sub template (1 μM) to provide maximum opportunity for template switching. As copy-choice recombination occurs during negative-strand synthesis (30) we also reasoned that a 60-fold excess of acceptor RNA over sym/sub template would be biologically relevant. Indeed, previous research has shown that negative strand synthesis is between 40–60-fold in excess of positive strand RNA synthesis during the early stages of RNA replication (31). The generation of a strand transfer product was dependent upon both the presence (Figure 4B) and sequence of the acceptor template. We initially investigated the suitability of an acceptor with a similar base composition (the ‘Random’ acceptor, Figure 4B) and demonstrated it was incompatible with formation of the transfer product. We extended this analysis to investigate the specific involvement of 3′ sequences in the acceptor by using each of three RNA molecules that were truncated by one, two or three nucleotides at the 3′ end, but were otherwise identical to the complementary RNA acceptor (Figure 5C). None could be incorporated into a transfer product.

### sym/sub analysis of fidelity polymerase variants

The development and optimization of a biochemically defined sym/sub recombination assay confirmed that the viral polymerase was the only protein required for formation of the strand transfer product. This assay therefore provides an experimental environment to investigate the direct influence of polymerase on strand transfer. Using the optimized conditions already established (Arnold *et al*., manuscript in preparation) we investigated the formation of the sym/sub strand transfer product by G64S, H273R and K359R polymerase fidelity mutants by quantifying the production of the strand transfer product 30 min after addition of acceptor template and nucleotides to the reaction. The high fidelity G64S polymerase variant generated 1.8-fold less of the strand transfer product compared to wild type at 30 min. In contrast, the H273R low fidelity variant increased template transfer product 3-fold. Strikingly, the generation of the strand transfer product by the K359R polymerase was so low that accurate quantification over a 30 minute time course was not possible (Figure 5A and B).

### Sequence analysis of the strand transfer product

The observations in both CRE-REP and sym/sub assays indicated that polymerase fidelity was a key determinant of strand transfer efficiency. The demonstration that the majority of the strand transfer products were of a minimum uniform size suggested there may be some sequence specificity to the *in vitro* recombination event, presumably involving one or other of the two limited regions of complementarity with the primary extension product (Figure 4A). In order to gain insight into the strand switching event we isolated the transfer product and subsequently cloned and sequenced the RNA species. Over 100 sequences were obtained, analysis of which resulted in two striking observations (Table 1). Firstly, the template switch occurred at the 3′ end of the acceptor template not at the internal region of complementarity. Secondly, a significant number of misincorporations or non-templated nucleotide additions occurred at or near the junction of the sym/sub donor and acceptor templates. Many of these involve the addition of C nucleotides, thereby increasing the potential for C–G base pairing with the 3′ end of the acceptor template. Only one of the sequences obtained showed no evidence of misincorporation or non-templated base additions. These observations support the role of polymerase fidelity in strand transfer and, together with the failure to observe a strand transfer product with random or truncated acceptor templates (Figures 4B and 5C), indicate that minimal regions of sequence complementarity are required for recombination *in vitro*.

## DISCUSSION

Recombination in mammalian positive sense RNA viruses, such as poliovirus, is a relatively poorly characterized mechanism responsible for the generation of extensive genetic variation in the progeny of co-infections. In a recent study we demonstrated that replicative recombination (i.e. in which both parental viruses are replication-competent, to distinguish it from a distinct non-replicative process that has also been described (16,17)) is a biphasic process that involves distinct strand-transfer and resolution events (11). Both phases are necessary for the generation of genome-length recombinants that circulate in the population (7,10). The CRE-REP assay developed in our previous study enabled both viral and cellular determinants of these phases to be investigated. However, being cell-based, it did not allow the influence of the viral polymerase to be defined and studied in isolation. Furthermore, recombination was only quantifiable within a limited region of the genome. We address both these restrictions here. We describe a modified CRE-REP assay in which recombination throughout the region encoding the viral non-structural proteins can be analysed. In addition, we show that recombination can be recapitulated in a defined biochemical system in which the only protein present is the viral polymerase. We use these two strategies to investigate the role of RNA dependent RNA polymerase fidelity and nucleotide turnover rate in the genetic recombination of poliovirus.

We had previously demonstrated that a G64S high fidelity polymerase variant significantly reduced (∼20-fold) the recombination rate observed in the CRE-REP assay. We extended this study by investigating the influence of K359R and H273R polymerase mutants, which are reported to exhibit higher and lower fidelity than the native enzyme respectively (26,27). In all instances, the relevant polymerase mutation was present in both the donor (sub-genomic replicon) and acceptor (SL3-derived) templates. In contrast to the G64S results, the presence of the H273R mutation led to at least a 3-fold increase in recombinant yield (Figure 1C), suggesting that low fidelity increases the rate of template switching by the viral polymerase, a result in agreement with a recent study in alphaviruses (15). Both the G64S and H273R variations have little impact upon the replication of poliovirus or sub-genomic replicons in cell based assays (27,32) indicating that fidelity alone may account for the changes in recombination rates measured in the CRE-REP assay. With the K359R mutation we were unable to detect any intra- or intertypic recombinants using the CRE-REP assay under conditions previously shown to yield viable progeny from G64S-containing templates (Figure 1C). Since the K359R polymerase has a lower fidelity than the G64S enzyme (26), fidelity alone cannot explain the different phenotypes of these enzymes in this assay. However, the K359R mutation also catalyses nucleotidyl transfer at 10% the rate of the unmodified enzyme (25,26), an observation we verified in luciferase assays using poliovirus type 1 or type 3 sub-genomic replicons (Figure 1A and B), which could also influence the recombination process. If there was a directly proportional relationship between replication rate and recombinant yield, we would have expected to detect recombinants in the intratypic CRE-REP assay (where the native enzyme generated ∼10^3^ pfu/ml) even if they were undetectable in the intertypic assay which is ∼20-fold less sensitive. However, when investigated (Figure 1C), the absence of recombinants in the intratypic CRE-REP assay by K359R-containing genomes, indicates that the yield was at least 3log_10_ lower, implying that replication rate and polymerase fidelity may exert a cumulative influence on recombination. Previous studies have already indicated that there is a direct relationship between elongation rate and polymerase fidelity (26,33). Due to the pleiotropic effect of mutations such as K359R on the activities of the polymerase it is difficult to determine the relative importance of these two polymerase phenotypes on recombination. In an attempt to achieve this we have investigated the consequences of adding the mutagen ribavirin on recombinant recovery in the CRE-REP assay primed with donor and acceptor RNAs bearing the high-fidelity G64S mutation (which exhibits similar processivity to the native enzyme (26)). Ribavirin at 600μM enhanced the yield of recombinants from either the intertypic or intratypic CRE-REP assay 2- to 3-fold (Supplementary Figure S4) providing additional confirmation that fidelity *per se* is a key determinant of the template switching events in recombination. Ribavirin at a similar concentration could not rescue recovery of recombinants from CRE-REP assays primed with templates bearing the K359R polymerase mutation (data not shown).

We reasoned that increasing the region within which recombination could occur in the CRE-REP assay might compensate for the reduced replication of the K359R polymerase mutant. We tested this by further separating the lesions that rendered the donor and recipient templates incapable of alone generating viable progeny, which we achieved by introducing a synthetic variant of the CRE to the 3′ NCR. In doing this, we demonstrated that the poliovirus CRE is functional in the 3′ NCR, 2.9 kb away from the native location and 6.8 kb distant from the 5′ NCR location previously reported (21). The robust replication of RLucΔCRE_3′CRE (Figure 2A and B) convincingly demonstrates the positional independence of the poliovirus CRE, as has also been shown for foot and mouth disease virus (34). Using a donor template (Figure 3A and C) with the CRE in the 3′ NCR increased recombinant yield by ∼5.8-fold in the modified 3′-CRE-REP assay over the original assay. The increased yield of recombinants was approximately proportional to the increase in separation of the genetic lesions used for the selection (5.8-fold versus 4-fold respectively). We interpret this as indicating there were no significant recombination ‘hotspots’ within the non-structural coding region that were absent from the ∼1 kb recombination window in the original CRE-REP assay. This conclusion is supported by preliminary analysis of a limited panel of recombinants from the 3′CRE-REP assay which indicated they were distributed throughout the region encoding the viral non-structural proteins (Supplementary Figure S3), with limited evidence of clustering at the 2C/3A boundary and the region encoding the C-terminus of the polymerase. We have previously suggested that the clustering of imprecise recombinants could be explained by functional constraints on the initial hybrid virus genome (11). The extended opportunity for recombination offered by the 3′CRE-REP assay will allow the influence of RNA structure, sequence identity and these functional constraints to be more fully examined in future studies.

Notwithstanding the increased yield of recombinants obtained from the 3′CRE-REP assay with the unmodified polymerase, inclusion of the K359R mutation completely inhibited the formation of detectable recombinant progeny (Figure 1C). This provides further support for this mutation having a non-recombinogenic phenotype, but does not help elucidate the role of reduced nucleotide incorporation and/or increased fidelity in achieving this behaviour.

A notable feature of the replication of the K359R polymerase is the apparent ‘stall’ in replication from around 2 to 6 h post-transfection. This was reported in a previous study (26) and also observed in sub-genomic replicons bearing this substitution (Figure 1A and B). During this period replication is occurring, but at a markedly reduced rate when compared to genomes bearing the native polymerase. After 6–8 h, replication gradually picks up, resulting in an overall yield ∼1log_10_ lower than the wild-type virus (26). The early events in genome replication include the formation and subsequent coalescence of membrane-anchored replication complexes (18,35). If one or more of these events is delayed, either directly by the action or interactions of the K359R polymerase, or indirectly because of reduced genome replication, then the yield of recombinants may be reduced. Nocodazole and cold treatment, which prevents microtubule re-polymerization, inhibits coalescence of RC and yield of recombinants (11,18). More compellingly, reversible stalling of virus replication using guanidine hydrochloride, also resulted in inhibition of the mobility of RCs (36). To try and discriminate between a direct influence of the K359R viral polymerase on the strand transfer event *per se*, and a defect in one or more of the cell-related events required for recombination, we investigated the process in a system in which the polymerase was the only protein present.

We exploited the well-characterised sym/sub assay that has previously been used to analyse the phenotype of the poliovirus polymerase in a biochemically-defined system. Briefly, an elongation complex is assembled on a 10 nt self-complementary template in the presence of ATP alone and the stalled extension relieved by addition of the remaining nucleotides. By inclusion of an acceptor template with limited complementarity to the primary extension product (Figure 4A and B) we were able to demonstrate the formation of a specific strand-transfer product which exhibited the expected reduced mobility, confirming that the polymerase alone is necessary for template switching during recombination. We used this assay to explore the influence of polymerase variants or the sequence of the acceptor template on the production of the strand-transfer product.

Our results strongly suggest that interactions of the donor and acceptor templates are important during recombination *in vitro*. An acceptor oligonucleotide with 6 nt. of complementarity (to the ‘strong stop’ extension product; underlined in Figure 4A) at its 3′ end was incorporated into the strand-transfer product, the length of which (Figure 4B) and subsequent sequencing (Table 1) indicated it is the position at which the polymerase switches template from donor to acceptor. The sequence specificity of this interaction was emphasised by the inability to generate a detectable strand-transfer product in the presence of an acceptor template with no complementarity (Figure 4B) or with acceptor templates truncated by 1–3 nucleotides at the 3′ end (Figure 5C). This analysis clearly demonstrates that the presence of two G nucleotides at the 3′ end of the acceptor—and complementarity between donor and acceptor template 3′ ends—was critical for generation of the transfer product.

**Table 1.** Sequence analysis of strand transfer products

#	Misincorporation	Untemplated	Sequence 5′-GCAUGGGCCC … -3′
24		+C	AUGC C AUGC AUGC UUGC N_0-3_
20		+CC	AUGC CC AUGC AUGC UUGC N_0-3_
2	G→C	+C	AUCC C AUGC AUGC UUGC N_0-3_
38	G→C		AUCC AUGC AUGC UUGC N_0-3_
14	(U/G)→C		ACC AUGC AUGC UUGC N_0-3_
4		+A(C/U)C	AUGC A(C/U)C AUGC AUGC UUGC
**1**			**AUGC AUGC UUGC**
1		ΔG	AUC AUGC UUGC

The first column indicates the total number of each sequence obtained, all of which were preceded with the core sym/sub sequence shown in the header line. Underlined nucleotides indicate misincorporations or untemplated additions (summarised in the indicated columns on the basis of the differences between the observed and expected [the latter highlighted in bold] sequences) at or near the junction of the sym/sub donor and acceptor sequences. N indicates the untemplated addition of any nucleotide, nucleotides in brackets indicate alternates, ΔG indicates a single nucleotide deletion.

Sequence analysis of the strand transfer product (Table 1) provides further insight to the importance of the two G nucleotides at the 3′ end of the acceptor. The majority of the sequences exhibited additional cytidine nucleotides at or near the recombination junction between the sym/sub donor and acceptor templates. Over 50% of the sequences included misincorporations, primarily of G to C, which would increase the sequence complementarity with the G dinucleotide at the 3′ end of the acceptor. In addition, an equivalent proportion (∼48%) of the sequences showed evidence of one or two non-templated C nucleotides at the junction. These presumably account for the reduced mobility apparent in some ‘strong stop’ products (Figures 4B and 5A) and would again increase the sequence complementarity between donor and acceptor molecules. Of the 104 transfer product sequences obtained, only one contained no sequence substitutions, additions or deletions from the expected product (5′-GCAUGGGCCCAUGCAUGCUUGC-3′). This level of variation has not been observed in the sequence of presumed recombination junctions in previous studies (1,37–41) or in our recent analysis using the CRE-REP assay ((11) and Bentley *et al*., manuscript in preparation), all of which involved analysis of replication-competent genomes. However, these analysed junctions were predominantly located within the polyprotein-coding region which, if disrupted, would be incompatible with virus viability. Over 60% of the strand transfer products sequenced (Table 1) contained additional nucleotides which would have resulted in the premature truncation of the open reading frame (ORF), and those in which the ORF remained intact contained additional amino acids, potentially poorly tolerated in the viral polyprotein. This strongly suggests that stringent functional selection of a diverse population of recombinant molecules may generate a much more limited subset of sequences which are observed in replication-competent viral populations.

Although the non-templated addition of nucleotides to the 3′ end of the donor was important in enhancing complementarity with the acceptor it does not alone account for the ability of the polymerase to generate a strand transfer product. The K359R polymerase generates reduced-mobility ‘strong stop’ products, indicative of this untemplated addition, but was unable to generate recombinant products in either the sym/sub or CRE-REP assays (Figures 1C and 5A). Additional polymerase fidelity mutants tested in the sym/sub assay also recapitulated the phenotypes previously characterized in the cell-based CRE-REP assay (Figures 1C, 4A and 5A). The low fidelity H273R polymerase yielded ∼3-fold more transfer product in the sym/sub assay and at least 3-fold more recombinants in the CRE-REP assay when compared with the unmodified polymerase. Similarly, in both the biochemical and cell-based assay the high fidelity G64S mutant polymerase exhibited a reduced yield, by ∼2-fold and ∼20-fold respectively (11). These results emphasise the relevance of the sym/sub recombination assay and allow the detailed dissection of the contributions of polymerase, the donor and acceptor molecules in the strand transfer event. This should provide important insights into the molecular mechanism of recombination in enteroviruses and, by extrapolation, other single-stranded RNA viruses. It may additionally help understand the processes involved in other polymerase/RNA interactions in which discontinuous RNA sequences are juxtaposed in the resulting product, for example during the generation of internally-deleted defective interfering genomes (42) or the involvement of the body and leader transcriptional regulatory sequences implicated in discontinuous subgenomic transcription in coronaviruses (43).

It is clear that the phenotype of the K359R mutant polymerase is of wider significance. This mutation lies within motif D, a conserved element present in all RNA dependent RNA polymerases, where the lysine acts as a general acid for nucleotidyl transfer (25,44,45). Not only does K359R attenuate poliovirus neurovirulence (26) but it is now shown that it also inhibits the frequency of template switching. Recombination between live attenuated Sabin vaccines and co-circulating species C enteroviruses is regularly observed and associated with paralytic disease (7,46,47). Once poliovirus eradication is achieved a K359R-containing variant could be considered as a suitable vaccine seed until global poliovirus vaccination programs are terminated.

Using the CRE-REP assay we have defined the recombination of enteroviruses as a biphasic process, with distinct recombination and resolution events. Of these, the first generates ‘imprecise’ junctions, effectively consisting of limited duplications within the genome. Sequence analysis of several hundred of these has failed to detect any sequence specificity to the process ((11) and unpublished). In contrast, the junctions resulting from the resolution event always contain ‘ambiguities’ where it is unclear whether the sequence at the junction is derived from the donor or recipient template. This implies that there may be a requirement for complementarity for resolution. Formally, recombination is a *trans* event involving the joining of separate molecules whereas resolution could occur either *in cis* or *in trans*. It is therefore interesting to note the sequence complementarity required for the generation of the strand-transfer product in the sym/sub assay which, by definition, must occur *in trans*. Further studies will determine whether the biochemically-defined sym/sub recombination assay is actually recapitulating the primary recombination or secondary resolution events described.

## Supplementary Material

SUPPLEMENTARY DATA
