# Supplementary material for: Biochemical and genetic analysis of the role of the viral polymerase in enterovirus recombination
Source: Nucleic Acids Res. 2016 Jun 17;44(14):6883–95. doi: 10.1093/nar/gkw567 (PMC5001610; doi:10.1093/nar/gkw567)
Supplement: SUPPLEMENTARY DATA [file supp_44_14_6883__index.html]

Biochemical and genetic analysis of the role of the viral polymerase in enterovirus recombination — Biochemical and genetic analysis of the role of the viral polymerase in enterovirus recombination — SUPPLEMENTARY DATA 

# Biochemical and genetic analysis of the role of the viral polymerase in enterovirus recombination

## SUPPLEMENTARY DATA

- SUPPLEMENTARY DATA
