# Supplementary material for: Biochemical and genetic analysis of the role of the viral polymerase in enterovirus recombination
Source: Nucleic Acids Res. 2016 Jun 17;44(14):6883–95. doi: 10.1093/nar/gkw567 (PMC5001610; doi:10.1093/nar/gkw567)
Supplement: SUPPLEMENTARY DATA [file supp_gkw567_nar-01016-v-2016-File011.pdf]

Supplementary Figure S1 – the addition of a functional synthetic CRE to the 3′NCR

RNA structure of the modified 3′ NCR predicted by MFOLD (http://unafold.rna.albany.edu/?q=mfold/RNA-Folding-Form) and redrawn for clarity. Numbering indicates the poliovirus type 1 reference sequence (Genbank Accession Number V01149.1). The sequence highlighted in red corresponds to Synth2, a partially-synthetic CRE previously published (21) engineered into a *Bss* HII site inserted immediately after the termination codon of the poliovirus polyprotein (underlined).

Supplementary Figure S2 – competition assays between unmodified type 1 poliovirus and a genome with the CRE relocated 3′NCR (FLCΔCRE_3′CRE)

The 8 mutations that inactivate CRE function (defined as the SL3 substitutions)(13) introduce a *Swa* I site to the genome. An RT-PCR product spanning the region of the 2C coding region encompassing the CRE was amplified from wild-type (FLC) poliovirus type 1 and from a virus in which the native CRE had been inactivated and a synthetic derivative inserted into the 3′NCR (FLCΔCRE_3′CRE), cut with *Swa* I and the products run on an agarose gel before ethidium bromide staining. The FLC product is uncut and the FLCΔCRE_3′CRE product cut into two pieces. The two viruses were mixed in 1:1, 10:1 or 1:10 ratios and the mix used to infect HeLa cells at an moi of 1. The virus-containing cell supernatant was passaged a further 5 times on HeLa cells, the RNA extracted from the first and last passage and used as template for the amplification of a product spanning the location of the native CRE. Over the course of the five passages neither virus predominates indicating that FLCΔCRE_3′CRE and unmodified poliovirus type 1 are broadly equivalent in replication fitness.

Supplementary Figure S3 – products of the extended 3′CRE-REP assay

Schematic diagram indicating the location of template switching from clonal recombinant isolates obtained for the 3′CRE-REP assay. Recombinants were recovered following co-transfection of murine L929 cells with RNA generated *in vitro* from pT7/SL3 (upper 'acceptor' genome) and pRLucΔ_3′CRE (lower 'donor' genome). The junction sequences of imprecise recombinants are indicated with solid lines, these contained in-frame duplications. Dashed lines indicate precise recombination products in which there are no duplicated sequences.

Supplementary Figure S4 – recombination of G64S templates in the CRE-REP assay is enhanced in the presence of ribavirin

Inter- or intratypic CRE-REP assays were primed with donor and acceptor templates bearing the G64S high-fidelity polymerase mutation in the absence (black bars) or presence of 600μM ribavirin (grey bars). Recombinant viruses were quantified by plaque assay and the results are expressed normalized to the yield of the same assay in the absence of ribavirin, with error bars indicating the standard deviation of three or more independent assays.

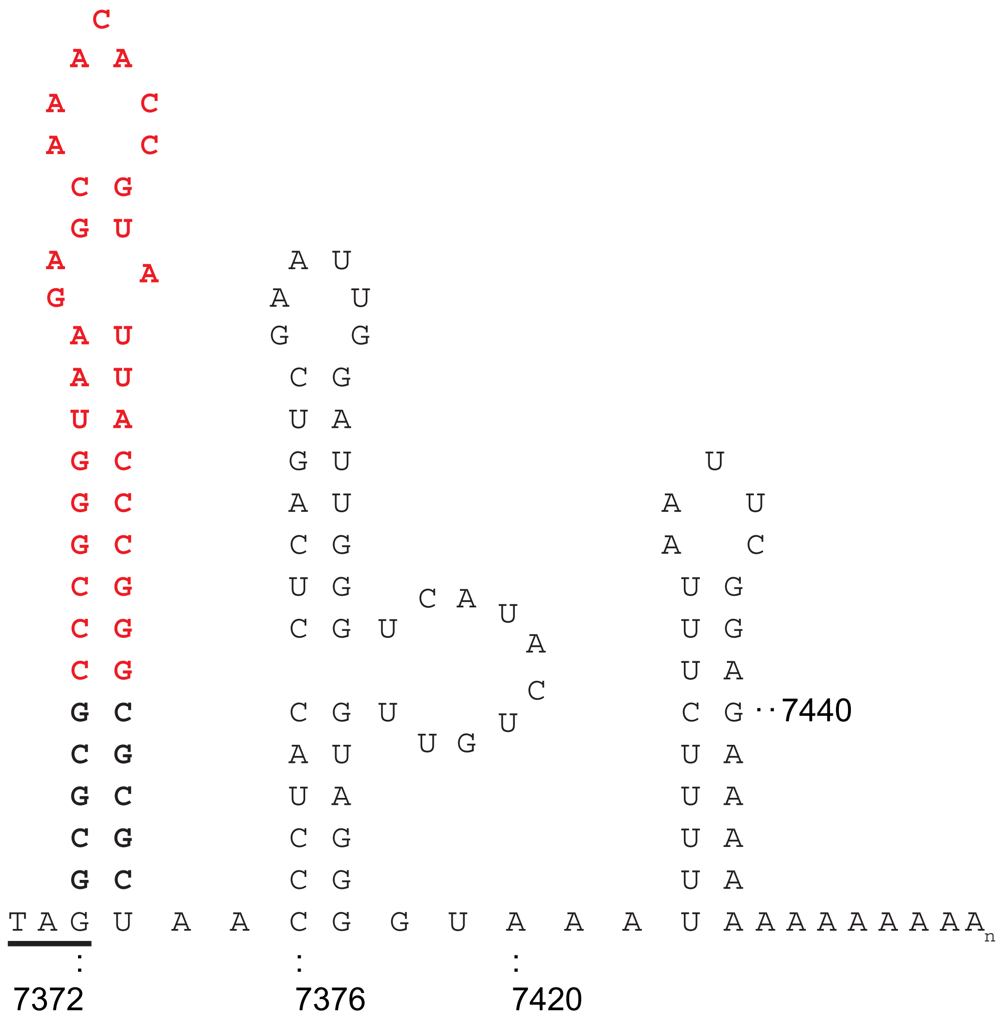

Woodman *et al.*, Supplementary Figure 1

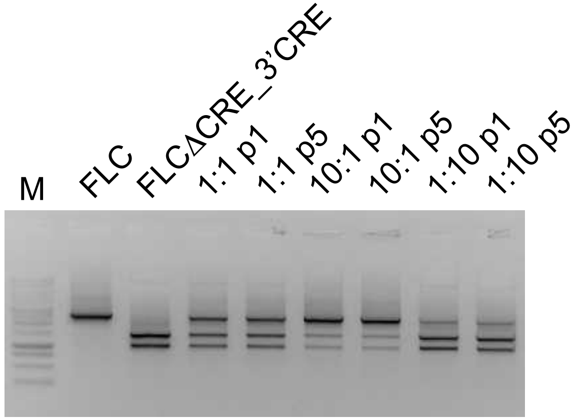

Woodman *et al*., Supplementary FIgure 2

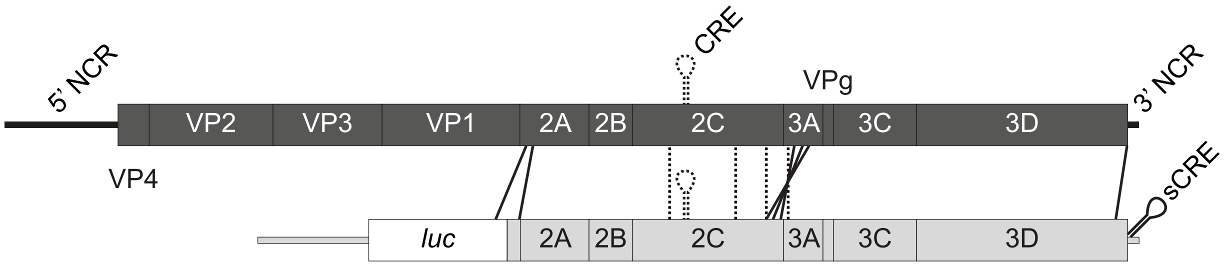

Woodman et al., Supplementary Figure S3

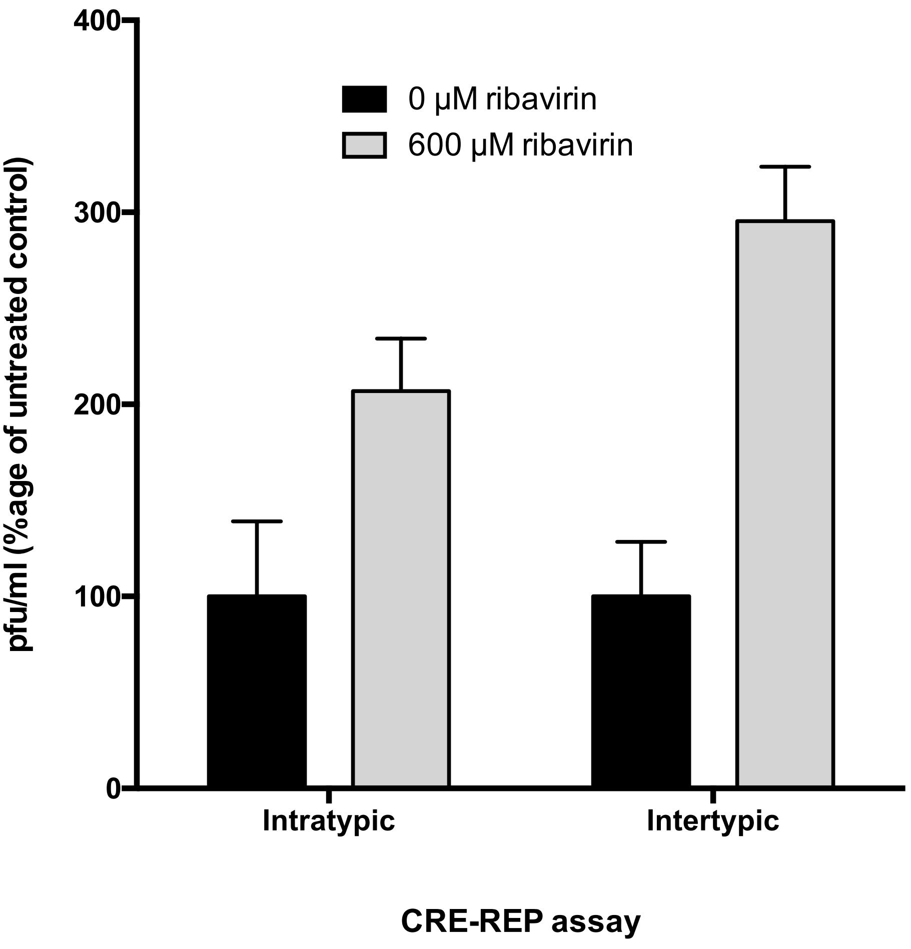

Woodman *et al*., Figure S4

Supplementary Table T1 – primers used in this study

Mutagenic primer	Sequence
PV1-G64S-F	CTCCAAGTACGTGTCTAACAAAATTACTGAAGTGGATGAG
PV1-G64S-R	CTCATCCACTTCAGTAATTTTGTTAGACACGTACTTGGAG
PV1-K359R-F	CTAACTATGACTCCAGCTGACCGATCAGCTACATTTG
PV1-K359R-R	CAAATGTAGCTGATCGGTCAGCTGGAGTCATAGTTA
PV3-G64S-F	CAATCTTCTCTAAGTATGTATCGAACAAGATCACTGAGGTGG
PV3-G64S-R	CCACCTCAGTGATCTTGTTCGATACATACTTAGAGAAGATTG
PV3-K359R-F	CATGACTCCGGCAGATAGGTCTGCCACTTTTGAGAC
PV3-K359R-R	GTCTCAAAAGTGGCAGACCTATCTGCCGGAGTCATG
CRE_MUT-F	CAACTATATCCAATTTAAATCCAAACACCGTATCGAACCAG
CRE_MUT-R	CTGGTTCGATACGGTGTTTGGATTTAAATTGGATATAGTTG
BSSHII-F	CATTTTAGGCGCGCTAACCCTACCTCAGTCGAATTGG
BSSHII-R	GGTTAGCGCGCCTAAAATGAGTCAAGCCAACGGCGGTAC
SYNTH_CRE-F	CGCGCCCGGGTAAGAGCAAACACCGTATTACCCGGG
SYNTH_CRE_R	CGCGCCCGGGTAATACGGTGTTTGCTCTTACCCGGG
PV1 H273R - FWD	CTAAACCACTCACAC**AGG**CTGTACAAGAATAAAACA
PV1 H273R - REV	TGTTTTATTCTTGTACAGCCTGTGTGAGTGGTTTAG
PV3 H273R - FWD	CTTAACCATTCACACAGGTGTTACAAAAACAAG
PV3 H273R - REV	CTTGTTTTTGTAACACCTGTGTGAATGGTTAAG
